# Bio-Based Pultruded CFRP Laminates: Bond to Concrete and Structural Performance of Full-Scale Strengthened Reinforced Concrete Beams

**DOI:** 10.3390/ma16144974

**Published:** 2023-07-12

**Authors:** Marina Machado, Mário Garrido, João P. Firmo, Adriana Azevedo, João R. Correia, João C. Bordado, Filipe Dourado

**Affiliations:** 1 CERIS, Instituto Superior Técnico, University of Lisbon, 1049-001 Lisbon, Portugal; mario.garrido@tecnico.ulisboa.pt (M.G.); joao.firmo@tecnico.ulisboa.pt (J.P.F.); joao.ramoa.correia@tecnico.ulisboa.pt (J.R.C.); 2 CERENA, Instituto Superior Técnico, University of Lisbon, 1049-001 Lisbon, Portugal; jcbordado@tecnico.ulisboa.pt; 3 S&P Clever Reinforcement Ibérica, 2845-408 Amora, Portugal; fdourado@sp-reinforcement.pt

**Keywords:** construction, sustainability, bio-polymers, composites, CFRP laminates, strengthening, RC structures

## Abstract

This paper presents an experimental study about the use of innovative bio-based pultruded carbon-fiber-reinforced polymer (CFRP) laminates for structural strengthening. The bio-based laminates were produced in the framework of an applied research project (BioLam) using a resin system with 50% (wt.%) bio-based content, obtained from renewable resources. In the first part of the study, their tensile and interlaminar shear properties were characterized and compared with those of conventional oil-based CFRP laminates. In the second part of the study, the bond behavior to concrete of both types of CFRP laminates applied according to the externally bonded reinforcement (EBR) technique was assessed by means of single-lap shear tests performed on CFRP-strengthened concrete blocks; the experimental results obtained from these tests were then used in a numerical procedure to calibrate local bond vs. slip laws for both types of laminates. The final part of this study comprised four-point bending tests on full-scale EBR-CFRP-strengthened reinforced concrete (RC) beams to assess the structural efficacy of the bio-based laminates; these were benchmarked with tests performed on similar RC beams strengthened with conventional CFRP laminates. The results obtained in this study show that the (i) material properties, (ii) the bond behavior to concrete, and (iii) the structural efficacy of the developed bio-based CFRP laminates are comparable to those of their conventional counterparts, confirming their potential to be used in the strengthening of RC structures.

## 1. Introduction

Approximately one third of global CO_2_ emissions are attributed to the construction industry. Increasing its efficiency and introducing innovative and more sustainable materials are key strategies to reduce the environmental impacts of this sector [1,2]. Fiber reinforced polymer (FRP) composites have gradually gained acceptance in construction due to several advantages they present over conventional materials [3,4,5,6]. However, their polymeric matrix is usually derived from petrochemical sources, generating significant environmental impacts and undesirable dependency from crude oil. In addition, during the past century, the price of both petrochemical monomers and oil-based products have suffered a high volatility due to increasing consumption, decreasing availability, and market instability [7]. Therefore, the use of renewable raw materials is typically pointed out as one of the solutions to reduce the environmental impacts of the construction industry, by reducing its dependence from petroleum [6,8,9,10,11].

Pultruded carbon-fiber-reinforced polymer (CFRP) laminates are often used in structural strengthening of civil engineering infrastructure, such as reinforced concrete (RC) bridges and buildings, and also in steel and timber structures. They have a typical fiber volume fraction of about 70% and their matrix is usually made of oil-based vinylester or epoxy resins. In this context, it is possible to improve the sustainability of these composite materials by using resins obtained from bio-based sources [8,10,11].

The most promising path for the development of biocomposites for structural applications seems to be the synthesis of polymers from renewable raw materials, i.e., biomass (either from agricultural waste or forestry-generated), from which vegetable oils, natural sugars, and natural polymers, such as lignin, cellulose, or hemicellulose, can be extracted in suitable biorefinery processes. This enables the FRP industry to improve the sustainability of its products by transitioning to bioresins derived from renewable raw materials, provided that these bio-based FRPs achieve comparable performance to those obtained with conventional polymeric resins [12,13].

One of the critical aspects that governs the structural efficiency of CFRP strengthening systems for reinforced concrete (RC) members is the bond behavior between the CFRP material and concrete. Although it is now well known that the bond behavior of conventional CFRP laminates to concrete relies mostly on the quality of the substrate (i.e., concrete properties and surface preparation method) and on the properties of the bonding adhesive, the material properties of CFRP laminates and especially the CFRP–adhesive interaction are key for the overall structural performance of the strengthening system. In this context, and in order to assess the feasibility of bio-based CFRP laminates for strengthening of RC structures, it is necessary to evaluate their bond behavior to concrete, namely, determining a set of properties that are still unknown for these bio-based composite products, more specifically, (i) their bond strength to concrete, (ii) the effective bond length, and (iii) the local bond vs. slip laws that describe the CFRP–concrete interaction and that are needed to numerically simulate CFRP-strengthened RC structures [14,15].

This paper presents an experimental study that aimed to assess the viability of using bio-based pultruded CFRP laminates for strengthening RC structures, by assessing their bond behavior to concrete through single-lap shear tests and their structural performance through four-point bending tests on CFRP-strengthened RC beams. To this end, tensile and interlaminar shear tests were performed on a bio-based CFRP laminate developed in the framework of the applied research project BioLam, and the mechanical performance of such a laminate was compared with that of conventional laminates. Then, the bond behavior to concrete of both types of CFRP laminates was assessed by means of single-lap shear tests performed on concrete blocks strengthened through the externally bonded reinforcement (EBR) technique; the experimental results obtained from these tests were used in a numerical procedure to calibrate local bond vs. slip laws. Finally, four-point bending tests on full-scale EBR-CFRP-strengthened RC beams were conducted to assess the structural efficacy of the bio-based laminates; as benchmark, similar tests were also performed on an unstrengthened RC beam and an RC beam strengthened with conventional EBR-CFRP laminates.

## 2. Overview of BioLam Project

The results presented in this paper are part of the BioLam project, whose main objective is to develop bio-based CFRP laminates with similar mechanical properties and structural performance as conventional CFRP laminates, made with vinylester or epoxy resins.

A first major step of the BioLam project was the in-house development of a bio-based unsaturated polyester (BUPE) resin, which has shown the necessary characteristics to be used in pultruded CFRP laminates, in terms of processability and mechanical performance. The BUPE resin, with a bio-based content of 50% (wt.%), was produced by synthesizing monomers (diacids and diols) derived from renewable raw materials, as detailed in Hofmann et al. [16]. The laminates were manufactured in a pilot pultrusion, which was successfully conducted using the BUPE resin; full details about the manufacturing process and the initial mechanical and thermo-mechanical properties of the bio-based laminates are provided in Hofmann et al. [17].

Besides the study presented in this paper, focused on the bond behavior to concrete and structural performance of CFRP-strengthened RC beams, the project also includes the assessment of the long-term durability of the bio-based CFRP strengthening system, including the effects of hygrothermal ageing (water immersion at temperatures of 20 °C, 35 °C, and 50 °C), elevated temperature, and UV radiation on the mechanical properties of the bio-based laminates and their bond to concrete. The project also includes a comparative life cycle assessment (LCA) of the bio-based CFRP laminates.

## 3. Materials and Methods

### 3.1. CFRP Laminates

The pultruded bio-based CFRP laminates were manufactured by S&P [17,18] using a bio-based unsaturated polyester resin obtained from renewable resources, which was recently developed at Instituto Superior Técnico [16] for use in FRP composite materials. The development of the bio-based unsaturated polyester prepolymer involved substituting some of the oil-based chemical components with bio-based alternatives, namely, fumaric acid, isosorbide, and 1,3-propanediol. Moreover, the amount of the (carcinogenic) reactive diluent styrene was decreased by partially replacing it with 2-hydroxy-ethyl methacrylate. The ratio of prepolymer to reactive diluents was 60:40 (wt.%). A detailed description of the development and characterization of this resin can be found in [16]. The laminates were reinforced with unidirectional carbon fiber rovings (Tenax^®^-E STS40 F13, supplied by Teijin) with 24,000 filaments, a diameter of 7 μm, a tensile strength of *f_u_* = 4300 MPa, and a modulus of elasticity of *E_f_* = 240 GPa.

Conventional CFRP laminates, produced by S&P as part of their regular product portfolio (C-laminate LA15002014), were used as a benchmark in this study; these conventional laminates were manufactured using a fully oil-based proprietary epoxy–vinylester resin and present the same carbon fiber reinforcement and content as the bio-based laminates. Both types of CFRP laminates were manufactured using a Pultrex pultrusion machine with a mold cross section of 20 mm × 1.4 mm; more information about the laminates’ composition and production method can be found in Hofmann et al. [17].

Tensile tests and interlaminar shear strength (ILSS) tests were used to assess and compare the mechanical properties of both types of laminates. Those tests were performed according to ASTM D638 [19], ASTM D7565/D7565M [20] and ASTM D5379/D5379M [21] standards, respectively, using universal test machines (UTM) from Instron, models 5989 (tensile tests) and 5982 (ILSS tests) (load capacities of 600 and 100 kN, respectively). DMA tests were performed to determine the thermomechanical response of both types of laminates and, more specifically, to compare their glass transition temperatures (*T*_g_). A TA Instruments DMA Q800 device was used, and the tests were performed according to the ASTM E1640 standard [22]. The specimens, with dimensions of 60 mm × 10 mm × 1.36 mm, were tested in a dual-cantilever configuration with a span length of 35 mm, a deformation amplitude of 15 μm, an oscillation frequency of 1 Hz, and a temperature sweep between −30 and 200 °C, at a constant heating rate of 2 °C·min^−1^. The values of *T*_g_ of the bio-based (79 °C) and conventional (80 °C) laminates were defined based on the onset of the storage modulus decay using a logarithmic scale, as specified in CEN/TS 19101 [23], and also on the tan δ peaks.

### 3.2. Structural Adhesive, Concrete, and Steel Reinforcement

For the four-point bending tests, three RC beams (rectangular cross section of 20 cm × 30 cm, length of 3.95 m) were cast using ready mixed concrete with an average cube compressive strength at the age of testing (cubes cured under the same conditions as the RC beams) of *f*_cm_ = 38 MPa and *f*_cm_ = 42 MPa, respectively, for the reference (i.e., unstrengthened) beam and for the two CFRP-strengthened beams. The RC beams were internally reinforced with longitudinal steel bars of class A400-NR, with diameters of 8 mm and 10 mm at the top and bottom layers, respectively, with two bars in each layer. Table 1 presents the elastic modulus, *E*_s_, yield stress, *f*_y_, and ultimate stress, *f*_u_, of the bars. The relatively low longitudinal reinforcement ratio adopted in the RC beams (*ρ* = 0.85%) was aimed at simulating a typical scenario where flexural strengthening is needed. The shear reinforcement consisted of steel stirrups (same class) with a diameter of 8 mm spaced at 15 cm; it was designed to prevent a shear failure of the CFRP-strengthened RC beams.

The CFRP–concrete interface is responsible for the stress transfer between the concrete substrate and the CFRP laminates, and it is typically materialized by means of a structural adhesive layer [24]. In this study, a conventional epoxy-based adhesive provided by S&P, with the commercial designation S&P-HP 220, was used to bond the CFRP laminates to the concrete substrate. Its properties, determined in Firmo et al. [25], are as follows (average ± standard deviation): shear strength, *τ*_u_ = 25.1 ± 1.5 (MPa); shear modulus, *G* = 4611.1 ± 565.9 (MPa); tensile strength, *σ*_u_ = 16.6 ± 1.9 (MPa); tensile modulus, *E* = 8205.3 ± 290.1 (MPa).

The concrete blocks used for the single-lap shear tests (with dimensions of 20 cm × 20 cm × 35 cm) were cast with premixed structural dry concrete with the commercial designation “SECIL Betão-S” using a water/cement ratio of 0.65, according to the manufacturer’s recommendations. The average value of the 28-day cube compressive strength was *f*_cm_ = 37 MPa, corresponding to a strength class of C25/30.

### 3.3. Bonding Procedures

Before bonding the CFRP laminates, the concrete substrates (concrete blocks and RC beams) were prepared in a similar manner: the concrete surface was lightly abraded until the aggregates became visible to increase roughness and enhance adhesion, and any remaining debris were removed from the substrate using compressed air. The CFRP laminates were cleaned with acetone to eliminate existing impurities. A thin layer of adhesive was directly applied on the concrete surface and the laminates were then positioned accordingly. The excess adhesive was removed using a specific thickness setter tool, resulting in a final adhesive layer of approximately 2 mm, as recommended by the manufacturer. The edges of the bonded area were finished with a 45° bevel to minimize stress concentrations.

**Figure 1:**
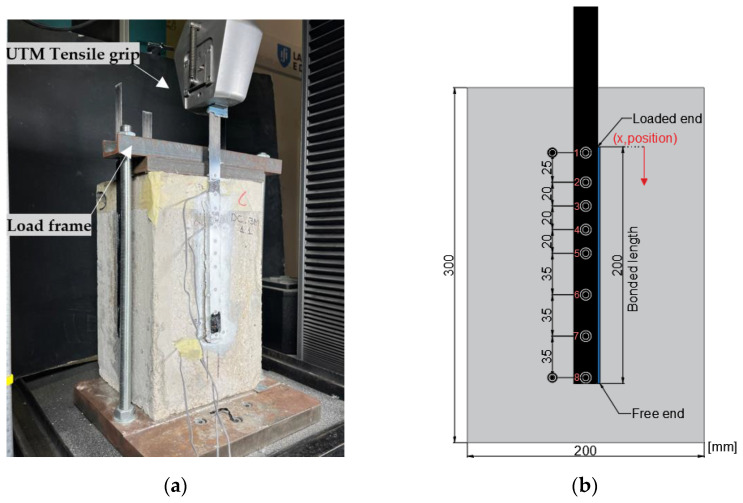
Single-lap shear tests: (**a**) overall view of test setup, and (**b**) bonded length and position of targets for VE (circles, numbered 1 through 8).

**Figure 2:**
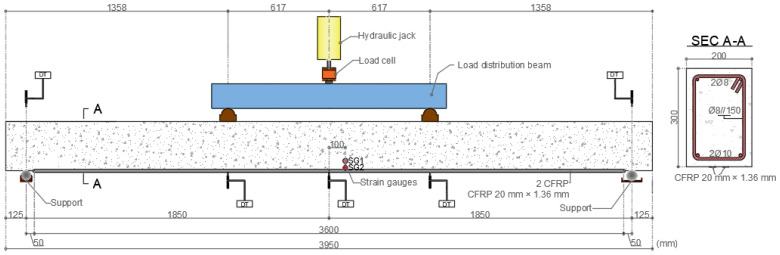
Four-point bending test configuration (**left**) and cross section of CFRP-strengthened RC beam (**right**) (DT—displacement transducer; SG—strain gauges bonded to the CFRP laminates).

Regarding the specimens used for the single-lap shear tests, four laminates were bonded on each concrete block, one at the center of each lateral surface (all aligned with the casting direction); a bonded length of 200 mm was adopted for all specimens (Figure 1), corresponding approximately to the anchorage length determined from different guidelines (e.g., 204 mm according to ACI 440 [26]). For the flexural tests of the RC beams, two CFRP laminates with a total length of 3.6 m were bonded to the bottom soffit of the beams, with a lateral spacing of 40 mm center-to-center (Figure 2). All specimens were stored in laboratory conditions (indoor, temperature and moisture not controlled) for a minimum duration of two weeks before testing, to guarantee an adequate curing of the adhesive as per the recommendations of the manufacturer.

### 3.4. Single-Lap Shear Tests

The single-lap shear test method was used to assess the bond behavior to concrete of both the conventional and the bio-based CFRP laminates; a minimum of three specimens were tested per type of laminate. Figure 1a shows an overview of the test setup; the concrete blocks were placed in a steel loading frame (made of two thick steel plates and two threaded steel rods) that was connected to a UTM from Instron, model 5982 (load capacity of 100 kN). Before the tests, the blocks were aligned using a laser level to minimize any eccentricities between the loading and longitudinal directions of the CFRP laminates. Then, the specimens were loaded in tension at a speed of 0.4 mm/min [19,20].

The following parameters were measured during the tests: (i) the load and crosshead displacement of the UTM; (ii) the slip at the beginning (loaded end) and at the end (free end) of the bonded length of the laminates, as well as at 6 additional intermediate positions of the bonded length (positions shown in Figure 1b), and (iii) the average axial strains along the bonded length of the CFRP laminates. The above-mentioned slip values and axial strains were measured using a videoextensometer (VE, camera FLIR BFS-U3-51S5M-C; lens Fujinon HF25SA-1; software from MatchID—v2022.2) that monitored the positions of targets marked on the specimens. As illustrated in Figure 1b, the targets were marked along the CFRP laminates and also on the concrete surface (next to the loaded and free ends of the laminates) allowing us to measure the relative displacement (i.e., the slip) between the laminates and the concrete, whereas the strains of the laminates were calculated through the variation of the position of consecutive pairs of targets (which provided the average strain between each pair of targets).

Experimental shear stress (*τ*) vs. slip (*s*) curves (also known as bond vs. slip curves) were computed based on the VE readings, by using the average strains between pairs of targets. Considering an elastic behavior of the CFRP, the average shear stress between two adjacent strain values (${\bar{\tau }}_{i+1/2}$) was obtained using Equation (1) that considers the difference between those strains (*ε_i_*_+1_ − *ε_i_*, at positions $x_{i+1}$ and $x_{i}$; *x* axis plotted in Figure 1b), and where *E*_t_ is the tensile modulus of the CFRP laminates, and *A*_f_ and *b*_f_ are, respectively, their cross-section area and width. The corresponding average slip values between positions $x_{i+1}\;$ and $x_{i}$ were computed from the VE readings.

(1) $${\bar{\tau }}_{i+\frac{1}{2}}=-\frac{E_{t}{\times A}_{f}\times \left(\varepsilon_{i+1}-\varepsilon_{i}\right)}{b_{f}\times (x_{i+1}-x_{i})}$$

### 3.5. Flexural Tests of Full-Scale RC Beams

To assess the structural effectiveness of the bio-based laminates, four-point bending tests were performed on three types of full-scale RC beams (as mentioned, with a length of 3.95 m and a cross section of 20 cm × 30 cm): (i) a reference (i.e., unstrengthened) beam, (ii) a beam strengthened with conventional CFRP laminates, and (iii) a beam strengthened with bio-based CFRP laminates. Figure 2 depicts a schematic view of the test setup.

The beams were monotonically loaded up to failure (using a hydraulic jack with 500 kN of capacity) at an average speed of 0.50 kN/s (until the yielding of the bottom steel rebars). The applied load was measured using a load cell (with capacity of 400 kN) and the vertical displacements of the beams at different locations (Figure 2) were monitored with electrical displacement transducers (DT, from TML, model *CDP-100*, precision of 0.01 mm). The axial strains of each CFRP laminate were measured at the midspan section with electrical strain gauges (from TML, model *FLKB-6-11-3LJC-F*).

## 4. Results and Discussion

### 4.1. CFRP Laminates Properties

**Figure 3:**
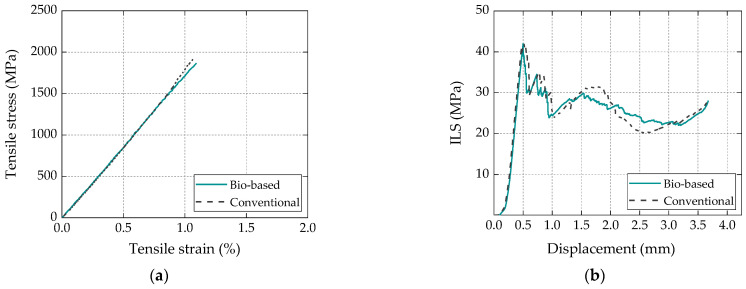
Behavior of representative specimens for bio-based and conventional laminates for (**a**) tensile and (**b**) ILS tests [17].

The mechanical and thermomechanical properties of the CFRP laminates, previously reported in [17], are provided here as they are relevant to the interpretation and understanding of the results of the current study. The tensile properties and ILSS of the CFRP laminates are summarized in Table 2, and Figure 3 depicts the behavior of representative specimens for both series obtained from tensile tests (Figure 3a) and interlaminar shear (ILS) tests (Figure 3b).

Figure 3 shows that the tensile and ILS responses of both types of laminates presented an overall similar behavior. Regarding the tensile properties of the bio-based CFRP laminates, when compared to their conventional counterparts, the average value of the tensile strength (*σ*_t_) was slightly higher (4%), the tensile modulus (*E*_t_) was slightly lower (4.5%), and the deformation capacity (*ε*_t_) was slightly higher (10%); however, these variations were generally within the experimental scatter. In terms of interlaminar shear properties, the average value of ILSS of the bio-based CFRP laminates was 10% lower than that of the conventional ones, indicating an appropriate (but slightly lower) adhesion between the bioresin and the carbon fiber rovings; this difference was meaningful with respect to the scatter of the results obtained for this property. The above-mentioned results confirmed the potential of the bio-based resin to be used in a high-structural-performance composite product.

Regarding the laminates’ thermomechanical response, the *T*_g_ values for the bio-based and conventional laminates defined based on the onset of the storage modulus decay (using a logarithmic scale) were 79 °C and 80 °C, respectively, i.e., they virtually matched. The *T*_g_ values for the same materials, but defined based on tan δ peaks, also presented similar values, 140 °C and 135 °C, respectively, for the bio-based and conventional laminates, confirming that both types of CFRP laminates exhibited a comparable thermomechanical response.

### 4.2. Single-Lap Shear Tests

#### 4.2.1. Load vs. Displacement/Slip Curves

**Figure 4:**
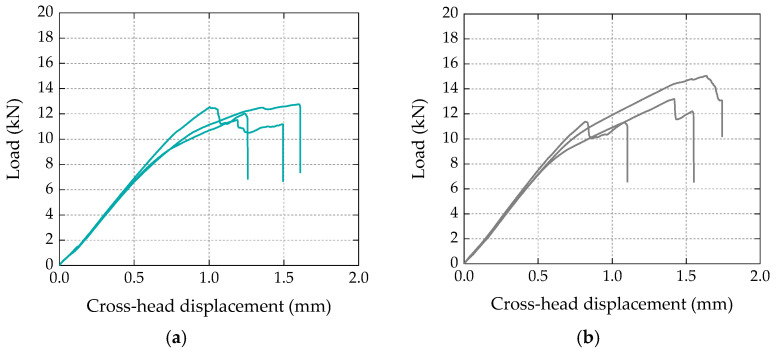
Load vs. cross-head (machine) displacement curves obtained in single-lap shear tests with (**a**) bio-based and (**b**) conventional CFRP laminates.

Figure 4 shows the load vs. crosshead displacement curves obtained for three specimens of each test series (in arbitrary order); in both types of specimens, the curves can be divided into three main stages: (i) a first stage up to loads of 7–8 kN, with an approximately linear response; (ii) a second stage characterized by a lower overall slope (i.e., stiffness), exhibiting a progressive stiffness reduction for load values approaching the maximum load, followed by (iii) a final stage presenting an abrupt load reduction (or, in some specimens, a two-step load reduction) corresponding to the failure of the bonded connection. In terms of bond strength (i.e., maximum loads), both types of specimens presented similar average values, with 12.3 kN vs. 13.2 kN for the bio-based and conventional laminates, respectively. The slightly higher (7%) bond strength of the specimens strengthened with conventional laminates was within the experimental uncertainty; the coefficient of variation (CoV) was 5% and 14%, respectively, for the test series with bio-based and conventional laminates.

**Figure 5:**
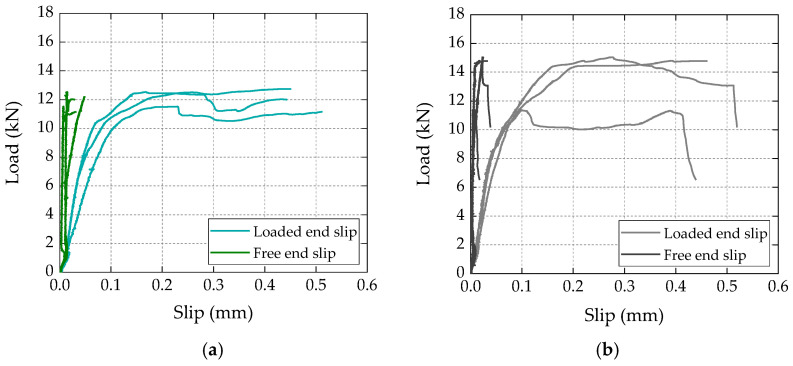
Load vs. slip curves of representative specimens with (**a**) bio-based and (**b**) conventional CFRP laminates.

Figure 5 presents the load vs. slip curves, for three different specimens (in arbitrary order), measured at the free and loaded ends of the laminates for both test series. Regarding the curves obtained at the free end of the laminates, both series presented negligible slip values (less than 0.05 mm); this result confirmed that for both conventional and bio-based laminates, the adopted bonded length (200 mm) was higher than the minimum anchorage length necessary to maximize the capacity of the bonded connections; as confirmed in the next section, this result showed that, for the materials and geometry of the specimens used in this study, the effective bonded length was shorter than (or, at the limit, equal to) the adopted bonded length.

Concerning the load vs. slip response at the loaded end of the CFRP laminates, the curves from both test series presented a similar behavior, with an initial linear stage (up to around 7–8 kN), followed by a progressive stiffness reduction up to the maximum load, after which the curves exhibited a load stabilization stage (or with slight load reductions), with increasing slip values up to failure; this last stabilization stage of the load vs. slip curves at the loaded end (well documented in the literature, e.g., [26,27,28]) is related to the damage progression from the loaded end towards the free end of the CFRP–concrete bonded connection. The axial strain measurements, presented ahead, provide further evidence of this damage progression.

#### 4.2.2. Failure Modes

**Figure 6:**
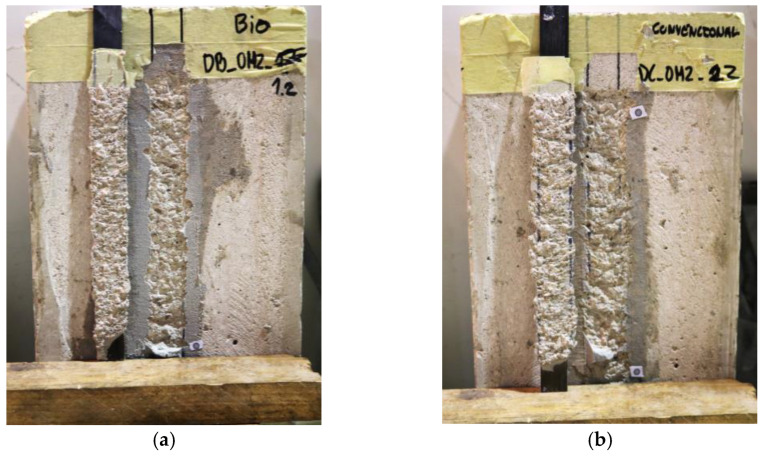
Representative failure modes of CFRP–concrete connection for specimens with (**a**) bio-based and (**b**) conventional CFRP laminates.

An overall similar behavior was also observed in the failure modes of specimens comprising both types of laminates; as shown in Figure 6, a cohesive failure within the superficial layer of the concrete was observed along the bonded length of both bio-based and conventional CFRP laminates. Future research is needed to assess/compare further the bond behavior of the bio-based laminates, in which different failure modes can occur (e.g., using higher concrete grades might impose interfacial debonding failures, given the slightly lower ILSS of the bio-based laminates, which can have a non-negligible impact on bond strength).

#### 4.2.3. CFRP Strain Distributions along the Bonded Length

**Figure 7:**
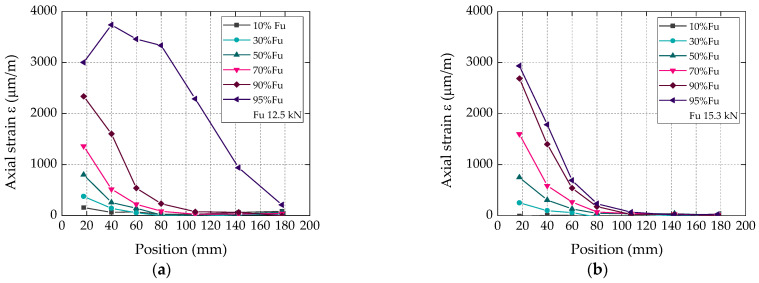
Axial strain distributions along bonded length (position *x* in Figure 1b) of representative specimens with (**a**) bio-based and (**b**) conventional CFRP laminates, for different fractions of the failure load.

Figure 7 shows the axial strain distributions along the bonded length for different percentages of the ultimate load (F_u_; position *x* marked in Figure 1b) of representative specimens with bio-based and conventional CFRP laminates for different fractions of the corresponding failure loads. As mentioned in Section 3.4, each data point of these plots corresponds to the average strain obtained between consecutive pairs of targets used for the VE. Overall, this figure confirms, again, a very similar behavior between specimens of the two test series: (i) for both types of laminates, and for load levels lower than 95% of the corresponding failure loads, the axial strain distributions presented the typical nonlinear development along the bonded length, with null strains close to the free end of the CFRP laminates and maximum strains at their loaded end; (ii) for higher load levels (i.e., in the brink of failure), the strains became non-negligible at the free end of the laminates and presented maximum values at a more central position of the CFRP-bonded length; these results (which could be traced more clearly in the bio-based laminate) were expected and are due to the damage progression from the loaded end towards the free end of the laminates.

#### 4.2.4. Experimental and Numerical Local Bond vs. Slip Laws

**Figure 8:**
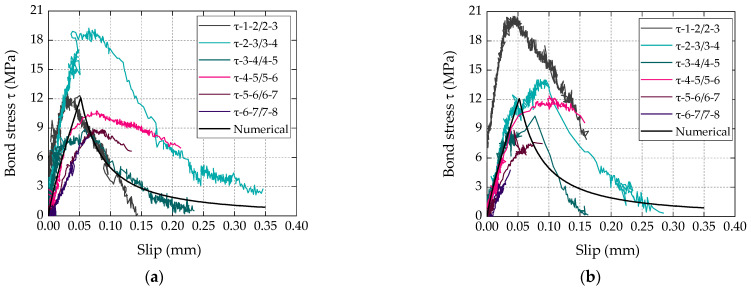
Experimental bond stress (*τ*) vs. slip curves with (**a**) bio-based and (**b**) conventional CFRP laminates.

The experimental bond vs. slip curves are illustrated in Figure 8, where each curve is representative of the average/local bond behavior between pairs of positions $x_{i+1},\;x_{i}$ along the bonded length (the procedure used to determine the shear stress vs. slip curves is explained in Firmo et al. [29]). The typical ascending and descending branches of the shear stress vs. slip response were captured, with the curves of both laminates presenting (as expected) an overall similar shape, with well-defined values of maximum shear stress.

It is worth highlighting the non-negligible scatter within each set of curves, which is a typical result in this type of experiment; it can be attributed to the local nature of the measurements, to cracking and to the intrinsic heterogeneity of the CFRP–concrete bonded connection (e.g., irregularity of the concrete surface, causing a nonuniform thickness of the adhesive, and hence a variation of the interface stiffness and strength along the bonded length).

Alongside the experimental shear stress vs. slip curves described above, local bond vs. slip curves were calibrated for both types of laminates using a numerical procedure previously adopted by the authors in [28]; this procedure allows the calibration of analytical expressions (described next) for the local bond laws that can be used/implemented in the numerical simulation of CFRP-strengthened RC structures.

The numerical procedure was developed in Matlab and used the load vs. slip data from the single-lap shear tests (i.e., results from Figure 5) to calibrate the parameters of the analytical local bond stress vs. slip relationships described by Equations (2) and (3), where $\tau \left(s\right)$ is the bond stress for a certain slip (*s*) value, $\tau_{m}$ is the bond strength, $s_{m}$ is the corresponding slip and α/α’ are parameters that define the shapes of the first and second branches, respectively.

(2) $$\tau \left(s\right)=\tau_{m}{\left(\frac{s}{s_{m}}\right)}^{\alpha }\;\;\;\;\;\;\;\;\;\;\;\;\;\;\;\;\;\;\;\;\;\;\;\;\;\;\;\;\;\;\;\;\;\;\;\;\;\;\;\;\;\;\;\;\;if\;s\;\leq \;s_{m}$$

(3) $$\tau \left(s\right)=\tau_{m}{\left(\frac{s}{s_{m}}\right)}^{-\alpha ’}\;\;\;\;\;\;\;\;\;\;\;\;\;\;\;\;\;\;\;\;\;\;\;\;\;\;\;\;\;\;\;\;\;\;\;\;\;\;\;\;\;if\;s>s_{m}$$

These analytical relations were previously (and successfully) used by Sena-Cruz and Barros [30] to simulate the bond behavior of CFRP–concrete bonded joints. The parameters of these laws were calibrated following the above-mentioned numerical procedure that simultaneously minimizes the area between the experimental and analytical load vs. slip curves, minimizing the differences between experimental and analytical maximum load and corresponding slip, and (initial) bond stiffness. Further information about this procedure and the numerical code can be found in Azevedo et al. [31]. In the present study, as negligible slip values were measured at the free end of the laminates, only the load vs. slip curves measured at the loaded end of the laminates were considered in the calibration procedure. The parameters defining the calibrated laws are summarized in Table 3 and the resulting local bond vs. slip laws are plotted in Figure 8, showing an overall good agreement with the experimental curves.

**Figure 9:**
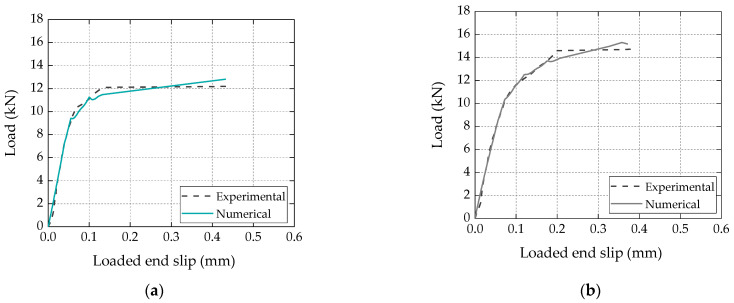
Comparison between numerical and experimental load vs. slip curves for (**a**) bio-based and (**b**) conventional laminates.

Figure 9 compares the numerical load vs. loaded end slip curve with the corresponding experimental curve (a representative specimen for each series). Again, it is clear that a very good agreement was obtained, further validating the numerical procedure and analytical bond vs. slip laws calibrated for both types of laminates.

### 4.3. Flexural Tests on Full-Scale Beams

**Figure 10:**
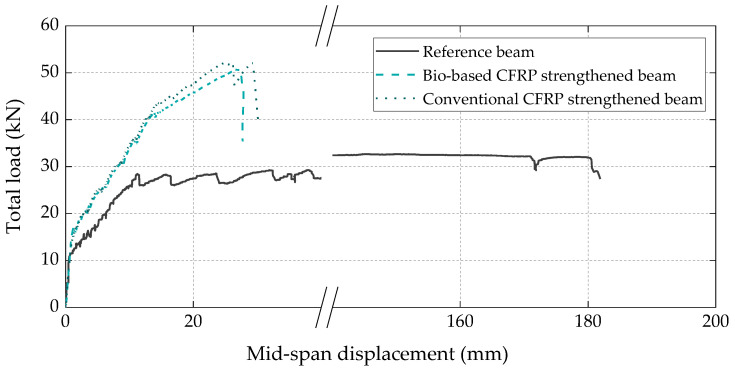
Total load vs. midspan displacement curves of all tested beams.

Figure 10 presents the load vs. displacement curves of the three full-scale RC beams. The behavior of all beams was consistent with the typical flexural response of conventional RC and CFRP-strengthened RC members, exhibiting the following three main stages: elastic, elastic-cracked, and elastoplastic. In the elastic stage, the strengthened beams exhibited similar cracking loads and stiffness when compared to the reference beam, whereas in the elastic-cracked stage, both CFRP-strengthened beams presented a slightly stiffer response than the reference beam due to the (more significant) contribution of the CFRP laminates. Regarding the elastoplastic stage of the strengthened beams, as expected, it was significantly shorter than that of the reference beam, as in the former beams the CFRP laminates detached relatively early during the steel yielding phase, which is typical of the EBR technique (the failure modes are detailed ahead). Both strengthened beams exhibited similar flexural strength, with failure loads of 51 kN and 52 kN, respectively, for the beams with bio-based and conventional laminates, which corresponded to a flexural strength increase of around 60% when compared to the reference unstrengthened beam.

**Figure 11:**
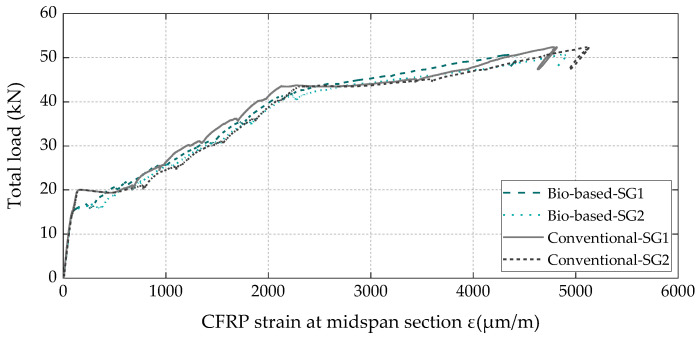
Total load vs. CFRP strain at the midspan section of both strengthened beams (note: 1 strain gauge per CFRP laminate, i.e., 2 per beam).

The total load vs. axial strain curves measured in the CFRP laminates at the midspan section (Figure 11) also exhibited the three main behavioral stages identified on the load vs. displacement curves (Figure 10) and, again, demonstrated that the bio-based laminates exhibited an equivalent structural efficacy to that of the conventional ones, with both attaining the same maximum strain (5.1‰) at the CFRP debonding instant.

**Figure 12:**
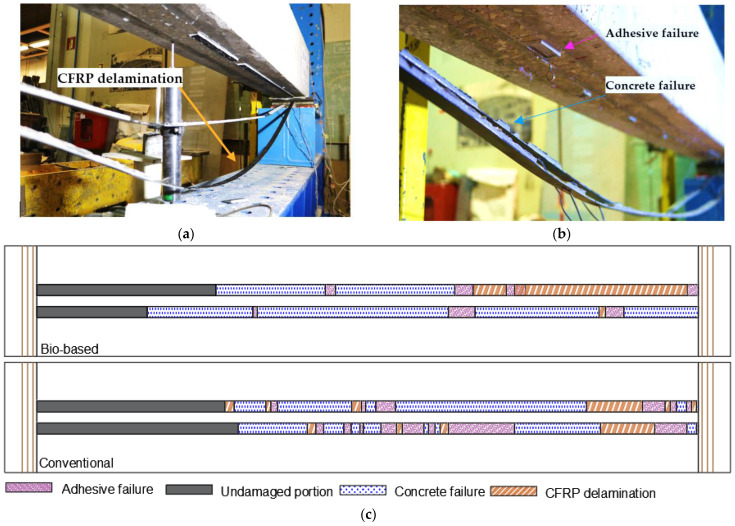
Local failure modes of CFRP strengthening system: (**a**) bio-based laminates; (**b**) conventional laminates; (**c**) schematic illustration along bonded length (not to scale).

In terms of failure modes, the reference RC beam collapsed due to concrete crushing after the bottom steel rebars yielded, whereas both strengthened beams failed due to the intermediate crack debonding of the CFRP laminates (initiated at the central zone) after the yielding of the bottom steel rebars (Figure 12).

Figure 12a,b show an overall view of the bottom surface of the strengthened beams after failure, and Figure 12c presents a schematic illustration of the different (local) failure modes along the CFRP strengthening system detected by visual inspection after the tests, identified as follows: (i) a cohesive failure of the superficial layer of concrete; (ii) a cohesive failure within the adhesive layer; and (iii) a CFRP delamination involving an interlaminar shear failure within the superficial layer of the laminates (identified by some carbon fibers attached to the adhesive); the length of the CFRP laminates that remained adhered to the substrate was identified as the “undamaged portion”. It is still worth mentioning that a failure at the concrete–adhesive (C-A) interface was not observed. Figure 12c shows that the beam strengthened with conventional laminates presented larger undamaged areas and a higher area of adhesive failure than the beam strengthened with the bio-based laminates; however, the latter exhibited a larger area of concrete failure and CFRP delamination, which may be attributed to a lower interfacial adhesion between the bio-resin and the carbon fibers, also reflected in the (slightly) lower ILSS mentioned in Section 4.1.

## 5. Concluding Remarks

This paper presented a pioneering study to assess the structural viability of using bio-based CFRP laminates for the flexural strengthening of RC members. In the first part of the study, these innovative composite materials were manufactured by pultrusion using a bio-based resin developed in-house, partially made from renewable raw materials. The material characterization tests showed that the tensile properties, the ILSS, and the *T*_g_ of the bio-based laminates were comparable to those of conventional (and commercially available) oil-based laminates.

The results of single-lap shear tests showed that the bio-based laminates also provided equivalent bond performance to conventional laminates, with the average bond strength of the former being only 7% lower than the latter. The local bond vs. slip laws curves were calibrated for both bio-based and conventional CFRP laminates, which can be used for modelling RC members strengthened with those laminates and installed according to the EBR technique.

The flexural tests on CFRP-strengthened RC beams confirmed the structural efficacy of the bio-based CFRP laminates; in fact, they performed similarly to their petroleum-derived counterparts, providing an identical (60%) flexural strength increase.

Overall, the results presented in this study demonstrate the feasibility of employing bio-based CFRP laminates to strengthen RC structural members, thus providing a more sustainable alternative to conventional oil-based counterparts.

## Figures and Tables

**Table 1:** Mechanical properties of steel bars used in RC beams (average ± standard deviation).

Diameter (mm)	*E*_s_ (GPa)	*f*_y_ (MPa)	*f*_u_ (MPa)
ϕ08	204.1 ± 3.5	489 ± 7.1	525.8 ± 7.9
ϕ10	208.2 ± 0.8	414 ± 6.9	584.7 ± 5.3

**Table 2:** Summary of mechanical properties of conventional and bio-based CFRP laminates (average ± standard deviation, coefficient of variation in square brackets, 5 samples for each material) [17].

Laminate Type	*σ*_t_ (MPa)	*E*_t_ (GPa)	*ε*_t_ (%)	ILSS (MPa)
Conventional	1947 ± 44 (2.3%)	174 ± 4.0 (2.2%)	1.3 ± 0.1 (7.7%)	45 ± 1.8 (4.0%)
Bio-based	2031 ± 171 (8.4%)	166 ± 4.0 (2.4%)	1.4 ± 0.2 (14%)	40 ± 1.1 (2.7%)

**Table 3:** Parameters of local bond vs. slip laws for bio-based and conventional CFRP laminates.

Type of Laminate	$\tau_{m}$	$s_{m}$	α	−α’
Bio-based	14.15	0.04	1.09	1.58
Conventional	12.12	0.05	0.84	1.38

## Data Availability

The data that support the findings of this study are available from the corresponding author, M.M., upon reasonable request.
